# Cholera

**Published:** 1860-10

**Authors:** 


*Cholera.*—An exchange states that this disease is traveling through Spain, and is now in Gibraltar. B.

